# Supplementary material for: Can non-pharmacological interventions reduce hospital admissions in people with dementia? A systematic review
Source: PLoS One. 2019 Oct 21;14(10):e0223717. doi: 10.1371/journal.pone.0223717 (PMC6802851; doi:10.1371/journal.pone.0223717)
Supplement: S1 Appendix — Appendix A-E. (DOCX) [file pone.0223717.s001.docx]

**Supplement 2**

Contents

[Appendix A: Search Terms 1](#_Toc21442084)

[Appendix B: Risk of Bias of Single Studies 4](#_Toc21442085)

[Appendix C: Reasons for exclusion 28](#_Toc21442086)

[Appendix D Supplementary References 29](#_Toc21442087)

# **Appendix A: Search Terms**

Database: Ovid MEDLINE(R) and Epub Ahead of Print, In-Process & Other Non-Indexed Citations and Daily <1946 to October 31, 2018>

Search Strategy:

--------------------------------------------------------------------------------

1 dementia/ or aids dementia complex/ or alzheimer disease/ or exp aphasia, primary progressive/ or exp dementia, vascular/ or diffuse neurofibrillary tangles with calcification/ or exp frontotemporal lobar degeneration/ or lewy body disease/ (133505)

2 alzheimer*.tw. (127134)

3 (dementia or demented).ti. (43977)

4 ((dementia or demented) adj4 (person* or senile or senility or elder* or subject* or adult* or patient* or people)).ab. (31001)

5 1 or 2 or 3 or 4 (192796)

6 hospitalization/ or patient admission/ or patient readmission/ or patient transfer/ (133661)

7 (hospitali#ation* or admission* or admit* or re-hospitali#ation* or rehospitali#ation* or readmit* or re-admit* or readmission* or re-admission*).mp. (508605)

8 Health Services/ (24022)

9 patient care management/ or "delivery of health care"/ (84555)

10 Case Management/ (9671)

11 Patient Care/ (9219)

12 Patient Care Team/ (61395)

13 "Quality of Health Care"/ (67827)

14 mental health services/ or community mental health services/ (48223)

15 Community Health Services/ (30149)

16 Health Services for the Aged/ (16894)

17 Nursing Care/ (28855)

18 Nursing Services/ (4067)

19 ((mental or health or community or nursing) adj3 service*).mp. (457511)

20 ((patient or elderly or community or nursing) adj3 car*).mp. (329081)

21 case-management.mp. (15838)

22 (service adj3 utili*).mp. (6130)

23 (health adj3 deliv*).mp. (114857)

24 or/6-23 (1311087)

25 5 and 24 (12808)

26 letter/ (1004201)

27 editorial/ (471469)

28 news/ (192201)

29 exp historical article/ (383792)

30 Anecdotes as topic/ (4725)

31 comment/ (738247)

32 case report/ (1905096)

33 (letter or comment*).ti. (134280)

34 or/26-33 (3973728)

35 exp Clinical Trial/ or exp Clinical Trials as Topic/ or Meta-Analysis/ or Meta-Analysis as Topic/ or Cross-Over Studies/ or exp Epidemiologic Studies/ (3108039)

36 (systematic review or meta-analys* or study or trial or cross-over or crossover or blind* or random*).mp. (8832934)

37 34 not (35 or 36) (3553728)

38 exp animals/ not humans/ (4510078)

39 exp Animals, Laboratory/ (824362)

40 exp Animal Experimentation/ (8851)

41 exp Models, Animal/ (520568)

42 exp rodentia/ (3061947)

43 (rat or rats or mouse or mice or rodent*).ti. (1294798)

44 or/37-43 (8773998)

45 25 not 44 (11557)

46 meta-analysis/ (93727)

47 exp meta-analysis as topic/ (17026)

48 (meta analy* or metaanaly* or metanaly* or meta regression).tw. (137758)

49 ((systematic* or evidence*) adj2 (review* or overview*)).tw. (163089)

50 (reference list* or bibliograph* or hand search* or manual search* or relevant journals).ab. (38456)

51 (search strategy or search criteria or systematic search or study selection or data extraction).ab. (45191)

52 (search* adj4 literature).ab. (53492)

53 (medline or pubmed or cochrane or embase or psychlit or psyclit or psychinfo or cinahl or science citation index or bids or cancerlit).ab. (171934)

54 cochrane.jw. (13967)

55 ((multiple treatment* or indirect or mixed) adj2 comparison).tw. (1606)

56 or/46-55 (363114)

57 exp randomized controlled trial/ or exp Randomized Controlled Trials as Topic/ (585294)

58 controlled clinical trial.pt. (92731)

59 randomi*.tw. (558783)

60 randomly.tw. (300309)

61 (clinical trials as topic or controlled clinical trials as topic).sh. (190341)

62 trial.ti. (189321)

63 or/57-62 (1242342)

64 56 or 63 (1503903)

65 45 and 64 (1537)

66 limit 65 to yr="2018 -Current" (113)

67 (201709* or 20171* or 2018*).ep. (980092)

68 ("2017/09*" or "2017/1*" or "2018/*" or "2019/*").ez. (1182041)

69 ("2017 Sep*" or "2017 Oct*" or "2017 Nov*" or "2017 Dec*" or "2017 09*" or "2017 1*" or "2018*").dp. (1471016)

70 67 or 68 or 69 (1556749)

71 65 and 70 (160)

72 66 or 71 (160)

Trials registries update search 2 November 2018- strategies and results

Dementia hospitalization avoidance

Clinical Trials.gov

Interventional Studies | ((dementia OR demented OR alzheimer OR alzheimers) AND (hospitalization OR hospitalisation OR readmission OR admission OR “mental health services” OR “health care services” OR “community services” OR “case management” OR “elderly care”)) | Adult, Older Adult | First posted from 09/01/2017 to 11/02/2018

Applied Filters: Interventional Adult (18–64) Older Adult (65+)

= 8 trials

WHO ( ICTRP portal)

Dementia and hospitalization OR dementia and hospitalisation OR dementia and admission OR dementia and readmission OR dementia and admit* OR dementia and rehospitalization OR dementia and rehospitalisation OR dementia and redmit* OR dementia and service* OR dementia and utilization OR dementia and ‘case management’ OR dementia and ‘health care’ OR dementia and ‘elderly care’ OR Alzheimer* and hospitalization OR Alzheimer* and hospitalisation OR Alzheimer* and admission OR Alzheimer* and readmission OR Alzheimer* and admit* OR Alzheimer* and rehospitalization OR Alzheimer* and rehospitalisation OR Alzheimer and readmit* OR Alzheimer and Service* OR Alzheimer and utilization OR Alzheimer*and ‘case management’ OR Alzheimer* and ‘health care’ OR Alzheimer* and ‘elderly care’

Registration 1/9/2017- 2/11/2018

Results= 45 trials

2 duplicates

# **Appendix B: Risk of Bias of Single Studies**

**Assessment of bias for Bass 2015 [1]**

| **Entry** | **Support for Judgement** | **Judgement** |
| --- | --- | --- |
| **Random Sequence Generation** | **Quote:** “one was randomly selected to deliver PDC and the other was deemed the comparison site that would deliver usual care (UC)”  **Comment:** Insufficient information | **Unclear risk.** |
| **Allocation Concealment** | **Quote:** “one was randomly selected to deliver PDC and the other was deemed the comparison site that would deliver usual care (UC)”  **Comment:** Predictable outcome based on the previous action. | **High risk.** |
| **Blinding of Participants and Personnel** | **Quote:** Not stated  **Comment:** From the design not possible to avoid. | **High risk.** |
| **Blinding of outcome assessment** (Hospitalisation) | **Quote:** Only reference is single line in the trial record [2], noted as “Single Blind (outcome Assessor)”  **Comment:** Insufficient information. | **Unclear risk.** |
| **Incomplete outcome data addressed** (Hospitalisation) | **Quote:**  **Comment:** Attrition rate similar between intervention and control, with similar reasons. | **Low risk.** |
| **Selective reporting** | **Quote:**  **Comment:** From reading trial record [2] and looking at other published work linked to the trial record [3-5], there are significant missing outcomes. | **Low risk.** |

**Assessment of bias for Bellantionio 2008 [6]**

| **Entry** | **Support for Judgement** | **Judgement** |
| --- | --- | --- |
| **Random Sequence Generation** | **Quote:** Not stated.  **Comment:** Not stated unclear. | **Unclear risk.** |
| **Allocation Concealment** | **Quote:** “The research assistant randomly assigned intervention or control status using sealed envelopes”  **Comment:** Probably true. | **Low risk.** |
| **Blinding of Participants and Personnel** | **Quote:** Not stated.  **Comment:** Not stated unclear. | **Unclear risk.** |
| **Blinding of outcome assessment** (Hospitalisation) | **Quote:** “A research assistant who was not part of the intervention team ascertained transition as well as reasons for transitions through weekly contact with the staff. The residents’ physicians and site staff made decisions regarding transitions. The same research assistant collected measures described below for study participants.”  **Comment:** Unclear if unaware of who was in the intervention. | **Unclear risk.** |
| **Incomplete outcome data addressed** (Hospitalisation) | **Quote:** Data not visible.  **Comment:** However not all data is presented. | **Unclear risk.** |
| **Selective reporting** | **Quote:** No pre-existing protocol exists.  **Comment:** Unclear. | **Unclear risk.** |

**Assessment of bias for Callahan 2006 [7]**

| **Entry** | **Support for Judgement** | **Judgement** |
| --- | --- | --- |
| **Random Sequence Generation** | **Quote:** “A random number table was used to classify the first physician as usual care if the table generated an even number or intervention if the table yielded an odd number. The second physician was then assigned the opposite status and the process was repeated until all physicians were randomized.”  **Comment:** Quasi random. | **High risk.** |
| **Allocation Concealment** | **Quote:** “if the table generated an even number or intervention if the table yielded an odd number. The second physician was then assigned the opposite status and the process was repeated until all physicians were randomized”  **Comment:** This is predictable. | **High risk.** |
| **Blinding of Participants and Personnel** | **Quote:** “Physicians were not informed of their randomization status and control physicians did not have access to the intervention. Members of the diagnostic team, the geriatric nurse practitioner, and patients and caregivers were blinded to the physician’s randomization status until the counselling session described above was completed and the patient consented to participate in the clinical trial and completed the baseline assessment.”  **Comment:** Participants not blinded or personal not blinded quote refers to before the start of the intervention. | **High risk.** |
| **Blinding of outcome assessment** (Hospitalisation) | **Quote:**” by telephone with interviewers who were blinded to the patient’s randomization status.”  **Comment:** Probably true. | **Low risk.** |
| **Incomplete outcome data addressed** (Hospitalisation) | **Quote:** Outcome data is available for all randomised participants.  **Comment:** All time points reported. | **Low risk.** |
| **Selective reporting** | **Quote:** “The patient and caregiver outcomes at all time points for both groups appear in **TABLE 4**.” There are no additional outcomes in the trial record [8]  **Comment:** Probably true. | **Low risk.** |

**Assessment of bias for Duru 2009 [9]**

| **Entry** | **Support for Judgement** | **Judgement** |
| --- | --- | --- |
| **Random Sequence Generation** | **Quote:** from [10] which describe method of the trial in more detail than Duru 2009 “computerized random-number generator operated by a study statistician”  **Comment:** Probably true. | **Low risk.** |
| **Allocation Concealment** | **Quote:** Not mentioned in either [9] or [10]  **Comment:** Not stated unclear. | **Unclear risk.** |
| **Blinding of Participants and Personnel** | **Quote:** from [10] which describe method of the trial in more detail than Duru 2009 “Participants were unaware of clinic randomization status at enrolment and at completion of the baseline survey and were not reminded of randomization status at follow-up” Assessors state not recorded.  **Comment:** Probably true. | **Unclear risk.** |
| **Blinding of outcome assessment** (Hospitalisation) | **Quote:** from [10] which describe method of the trial in more detail than Duru 2009 “we did not assess the extent to which abstractors were blinded to intervention status”  **Comment:** Likely problem. | **High risk.** |
| **Incomplete outcome data addressed** (Hospitalisation) | **Quote:** “We used an intent-to-treat  framework based on the initial randomization and limited the main analyses to patients with complete utilization data at baseline who survived for the entire study period and had follow-up data at 12 months, 18 months, or both.” A large proportion of people did not complete the study, no characteristics of those who left are included in the paper.  **Comment:** Unclear from the report. | **Unclear risk.** |
| **Selective reporting** | **Quote:** Would appear that all of the many outcomes registered in both papers [9, 10] have been reported on.  **Comment:** Probably done. | **Low risk.** |

**Assessment of bias for Eloniemi-Sulkava 2009 [11]**

| **Entry** | **Support for Judgement** | **Judgement** |
| --- | --- | --- |
| **Random Sequence Generation** | **Quote:** “the study nurse phoned a randomization center staff member who had not met the couples or seen their clinical records.”  **Comment:** Probably true. | **Low risk.** |
| **Allocation Concealment** | **Quote:** “Neither the study nurse nor the randomization center staff member could influence the process because the randomization assignments appeared in the program only after a couple’s name and participant number were inserted into it”  **Comment:** Probably true. | **Low risk.** |
| **Blinding of Participants and Personnel** | **Quote:** Not mentioned  **Comment:** Not stated unclear. | **Unclear risk.** |
| **Blinding of outcome assessment** (Hospitalisation) | **Quote:** “the study nurse was not blinded to treatment allocation in the follow-up data collection.”  **Comment:** Probably true. | **High risk.** |
| **Incomplete outcome data addressed** (Hospitalisation) | **Quote:** “Figure 1: 100% complete intervention”  **Comment:** Figure 1 contained hospitalisation data. | **Low risk.** |
| **Selective reporting** | **Quote:** No protocol, several elements not reported such as mortality.  **Comment:** Unclear. | **Unclear risk.** |

**Assessment of bias for Engedal 1989 [12]**

| **Entry** | **Support for Judgement** | **Judgement** |
| --- | --- | --- |
| **Random Sequence Generation** | **Quote:** “They were randomly allocated  to a day-care group and a control group”  **Comment:** Insufficient detail. | **Unclear risk.** |
| **Allocation Concealment** | **Quote:** They were randomly allocated  to a day-care group and a control group  **Comment:** Insufficient detail. | **Unclear risk.** |
| **Blinding of Participants and Personnel** | **Quote:** Not stated  **Comment:** Not stated unclear. | **Unclear risk.** |
| **Blinding of outcome assessment** (Hospitalisation) | **Quote:** Not stated  **Comment:** Not stated unclear. | **Unclear risk.** |
| **Incomplete outcome data addressed** (Hospitalisation) | **Quote:** “Drop-outs before follow-up were included in the analysis, according to the intention-to-treat approach.”  **Comment:** Probably done. | **Low risk.** |
| **Selective reporting** | **Quote:** Nil relevant.  **Comment:** Not clear. | **Unclear risk.** |

**Assessment of bias for Graff 2008 [13]**

| **Entry** | **Support for Judgement** | **Judgement** |
| --- | --- | --- |
| **Random Sequence Generation** | **Quote:** “A statistician not involved in the study carried out randomization” no additional information from previous reported paper [14].  **Comment:** Uncertain of procedure used. | **Unclear risk.** |
| **Allocation Concealment** | **Quote:** “Concealed envelopes were used to allocate the patients and these envelopes were opened by an independent secretary”  **Comment:** Probably true. | **Low risk.** |
| **Blinding of Participants and Personnel** | **Quote:** “Patients and care givers were aware of the treatment assigned.”  **Comment:** Probably true. | **High risk.** |
| **Blinding of outcome assessment** (Hospitalisation) | **Quote:** “The assessors (MT or MJLG) were  blinded to group allocation.” “Patients and care givers were asked before each assessment not to inform the assessors about the intervention. To check the success  or failure of the blinding after each measurement the assessors were asked if they had been told or knew for sure to which group each patient had been allocated.” From [14] “We tried to maintain masked conditions for assessment, however, which succeeded for 80% of the cases.”  **Comment:** Probably true. | **Low risk.** |
| **Incomplete outcome data addressed** (Hospitalisation) | **Quote:** “Of the 135 eligible patients randomised, three (one in the intervention group, two in the control group) with their care givers stopped the trial immediately after randomisation because they did not want to continue and they did not receive the study intervention. This left 132 patients with care givers for the intention to treat analysis9 and the cost effectiveness analysis.”  **Comment:** Probably done. | **Low risk.** |
| **Selective reporting** | **Quote:** Spread over multiple papers it’s unclear if all outcomes have been reported.  **Comment:** Unclear. | **Unclear risk.** |

**Assessment of bias for Kohler 2014 [15]**

| **Entry** | **Support for Judgement** | **Judgement** |
| --- | --- | --- |
| **Random Sequence Generation** | **Quote:** “Eligible patients were allocated to intervention or control group by the current status of their attending physician as a full member and associated member of dementia network, respectively.”  **Comment:** Probably true. | **High risk.** |
| **Allocation Concealment** | **Quote:** Nil additional to above.  **Comment:** Probably not done | **Unclear risk.** |
| **Blinding of Participants and Personnel** | **Quote:** No mention in paper.  **Comment:** Not stated unclear. | **Unclear risk.** |
| **Blinding of outcome assessment** (Hospitalisation) | **Quote:** No mention in paper.  **Comment:** Not stated unclear. | **Unclear risk.** |
| **Incomplete outcome data addressed** (Hospitalisation) | **Quote:** 11 more people dropped out of the study in the intervention side than the control side, there is no mention of these in the report, or any sensitivity analysis.  **Comment:** Variation in the groups is unclear given the available data. | **Unclear risk.** |
| **Selective reporting** | **Quote:** No protocol, outcome measure only listed in results section.  **Comment:** Not clear. | **Unclear risk.** |

**Assessment of bias for Laakkonen 2016 [16]**

| **Entry** | **Support for Judgement** | **Judgement** |
| --- | --- | --- |
| **Random Sequence Generation** | **Quote:** “Couples were randomly allocated using computer-generated random numbers.”  **Comment:** Probably true. | **Low risk.** |
| **Allocation Concealment** | **Quote:** “A person unrelated to the intervention telephoned a person at a randomization center who did not know the couples’ identities. The person read the names from a list in the order in which they had been assessed. Couples were randomly allocated using computer-generated random numbers.”  **Comment:** Probably true. | **Low risk.** |
| **Blinding of Participants and Personnel** | **Quote:** “Group facilitators performing the intervention knew whom they were treating but were unaware of the study endpoints. Study nurses, who were not part of the intervention staff, were not informed about the group allocation of participants, but participants were eager to share their experiences. Thus, study nurses could not be kept entirely blinded, although they did not know what occurred in the interventions for participants, nor were they co-investigators.”  **Comment:** Probably true. | **High risk.** |
| **Blinding of outcome assessment** (Hospitalisation) | **Quote:** No mention of this in the study.  **Comment:** Not stated unclear. | **Unclear risk.** |
| **Incomplete outcome data addressed** (Hospitalisation) | **Quote:** “Data were also collected during 24 months of follow up on all participants’ use and costs of health and social services.”  **Comment:** Probably done. | **Low risk.** |
| **Selective reporting** | **Quote:** Several elements from the trial registry [17] are not reported including GHQ-12 and Cornell depression score are not reported.  **Comment:** Evidence of selective reporting. | **High risk.** |

**Assessment of bias for Meeuwsen 2013 [18]**

| **Entry** | **Support for Judgement** | **Judgement** |
| --- | --- | --- |
| **Random Sequence Generation** | **Quote:** From previous report [19] “Randomisation will take place by computer”  **Comment:** Probably true | **Low risk.** |
| **Allocation Concealment** | **Quote:** From previous report [19] “the researcher is not able to predict randomisation outcome for a particular patient.”  **Comment:** Unclear how this is achieved. | **Unclear risk.** |
| **Blinding of Participants and Personnel** | **Quote:** From previous report [19] “After randomisation, both patient and physician need to be informed about the treatment a patient will receive during the study.”  **Comment:** Probably true. | **High risk.** |
| **Blinding of outcome assessment** (Hospitalisation) | **Quote:** From previous report [19] “Although we know from experience that blind assessment of the outcome may be difficult, we intend to keep the research assistants who are performing the measurements blind to the treatment a participant receives.”  **Comment:** Unclear if outcome of bias avoidance has been achieved. | **Unclear risk.** |
| **Incomplete outcome data addressed** (Hospitalisation) | **Quote:** “Eleven pairs (four in the memory clinic group) dropped out because they considered further participation to be too burdensome. One caregiver died, one caregiver did not fill out the questionnaires, and one caregiver was not present during the measurements without giving any reasons, all in the general practitioner group. In one patient in the general practitioner group the diagnosis of dementia was changed just after inclusion and was the reason for the patient and caregiver to withdraw from the study.”  **Comment:** Minimal subjects lost to follow up in this study with little difference in rationale between the groups. | **Low risk.** |
| **Selective reporting** | **Quote:** Reviewing the protocol [19] and the trial record [20] there are no unreported measures.  **Comment:** Probably true. | **Low risk.** |

**Assessment of bias for Pitkala 2013 [21]**

| **Entry** | **Support for Judgement** | **Judgement** |
| --- | --- | --- |
| **Random Sequence Generation** | **Quote:** "The randomization was performed by using computer-generated randomly allocated numbers received by telephone from a randomization center."  **Comment:** Probably true. | **Low risk.** |
| **Allocation Concealment** | **Quote:** "received by telephone from a randomization center. A study nurse called a person at a randomization center who did not know the identities of the potential participants. The study nurse read the names from a printed list in the order in which they had been assessed."  **Comment:** Probably true. | **Low risk.** |
| **Blinding of Participants and Personnel** | **Quote:** "caregivers did not know about the study hypothesis."  **Comment:** Insufficient evidence of blinding. | **High risk.** |
| **Blinding of outcome assessment** (Hospitalisation) | **Quote:** "The research staff was independent from the staff delivering the intervention, and the assessors were not coinvestigators and therefore did not know what was happening in the interventions."  **Comment:** Probably true. | **Low risk.** |
| **Incomplete outcome data addressed** (Hospitalisation) | **Quote:** "All randomized patients were included in the use and costs of services analyses (intention to treat)."  **Comment:** Probably true. | **Low risk.** |
| **Selective reporting** | **Quote:** Protocol [22] quoting secondary outcomes "Physical function (Barthel index), institutionalization, behavioural symptoms, falls, caregiver burden, cost-effectiveness, total mortality", care giver burden is not presented neither are behavioural symptoms.  **Comment:** Uncertain if measured certainly not presented. | **High risk.** |

**Assessment of bias for Sogaard 2014 [23]**

| **Entry** | **Support for Judgement** | **Judgement** |
| --- | --- | --- |
| **Random Sequence Generation** | **Quote:** “The randomisation was done with Stat Direct version 2.3.7. We used a random block size algorithm to prevent imbalance between the allocation groups.” From [24]  **Comment:** Probably true. | **Low risk.** |
| **Allocation Concealment** | **Quote:** “The allocation procedure was concealed for the DAISY project group and was conducted by an independent department in Rigshospitalet. At enrolment, a fax was sent to this department together with a participant code and information regarding stratification. Then the stratification was conducted, and the DAISY group received a fax with information regarding allocation status” From [24]  **Comment:** Probably true. | **Low risk.** |
| **Blinding of Participants and Personnel** | **Quote:** This was a “rater blinded” study, the participants and the doctors were aware of allocation.  **Comment:** | **High risk.** |
| **Blinding of outcome assessment** (Hospitalisation) | **Quote:** “The allocation code was concealed for the raters at follow-up visits and each rater could not rate a dyad more than once.” From [24, 25]; The raters were not involved in the intervention programme, and they were not employed in the same institutions as the study coordinators and counsellors. Patients and caregivers were instructed to try not to reveal which treatment arm they were in. The efficiency of concealment was checked using a questionnaire to the raters at the end of each follow-up visit. None of the raters visited the same patient-caregiver couple more than once.  **Comment:**. | **Low risk.** |
| **Incomplete outcome data addressed** (Hospitalisation) | **Quote:** Outcome data is available for all randomised participant after author correspondence.  **Comment:** All time points reported. | **Low risk.** |
| **Selective reporting** | **Quote:**  **Comment:** The hospitalisation data was only available on request to the author. | **Low risk.** |

**Assessment of bias for Schwarzkopf 2011 [26]**

| **Entry** | **Support for Judgement** | **Judgement** |
| --- | --- | --- |
| **Random Sequence Generation** | **Quote:** “The randomization was done by the statistics and data centre, usually one day before each training course when the full list of registered participants was available..” From [27].  **Comment:** Probably true. | **Low risk.** |
| **Allocation Concealment** | **Quote:** Not Explicitly stated in the paper or protocol. [27, 28]  **Comment:** | **Unclear risk.** |
| **Blinding of Participants and Personnel** | **Quote:** “The study is unblinded with respect to the GPs, the patients, and their caregivers.” From [27]  **Comment:** Probably true | **High risk.** |
| **Blinding of outcome assessment** (Hospitalisation) | **Quote:** “However, the assessment by  telephone interview after one and two years is done by students who are not informed to which study arm the patient belongs.”  **Comment:** Probably true | **Low risk.** |
| **Incomplete outcome data addressed** (Hospitalisation) | **Quote:** Outcome data is available for all randomised participant after author correspondence.  **Comment:** All time points reported. | **Low risk.** |
| **Selective reporting** | **Quote:**  **Comment:** The hospitalisation data was only available on request to the author. | **Low risk.** |

**Assessment of bias for Voigt-Radloff 2011 [29]**

| **Entry** | **Support for Judgement** | **Judgement** |
| --- | --- | --- |
| **Random Sequence Generation** | **Quote:** “The random allocation sequence was computer-generated with blocking by centre and groups of two persons, without stratification and in a ratio of 1:1 by a statistician from a distant site.”  **Comment:** Probably true. | **Low risk.** |
| **Allocation Concealment** | **Quote:** “The statistician emailed the individual allocation to COTiD or COTC exclusively to the site interventionist and stored the allocation list at his distant site which was not available to any study site staff. The interventionist scheduled treatment sessions, faxed records to the distant coordinating study centre and kept all documents strictly separated from any other site staff.”  **Comment:** Probably true. | **Low risk.** |
| **Blinding of Participants and Personnel** | **Quote:** “Since the numbers of home visits differed in the experimental and control groups, masking of patients and carers was not possible.”  **Comment:** Probably true. | **High risk.** |
| **Blinding of outcome assessment** (Hospitalisation) | **Quote:** “Assessors ‘blind’ for the group assignment, completed measurements at the patient’s home at baseline and at weeks 6, 16 and 26, and arranged a postal survey of carer questionnaires at week 52.” “However, study information did not include any preference for a special treatment ‘arm.’ Patients and carers were asked to give no information about their treatment package to assessors or study physicians. All study personnel were ‘blind’ for group assignment, except the interventionists. Agreement between the assessors’ estimation of group assignment and the actual group assignment was 61%, and thus slightly over the expected 50% of agreement by chance.”  **Comment:** Probably true. | **Low risk.** |
| **Incomplete outcome data addressed** (Hospitalisation) | **Quote:** “In the total sample of all randomised  participants (n¼141), two deaths of patients (both in the control group) and one death of a carer (in the COTiD group) were reported. In the COTiD group, 14 patients were admitted to hospital for an average of 15 nights, and 10 patients in the control group for an average of 18 nights.”  **Comment:** Probably true. | **Low risk.** |
| **Selective reporting** | **Quote:** “There was one protocol amendment before recruitment started. The Assessment of Motor and Process Skills was replaced by the PRPP, because the Assessment of Motor and Process Skills was not available in the German language within the planned schedule.” There appear to be no major differences between protocol [30] and this study.  **Comment:** Probably true. | **Low risk.** |

**Assessment of bias for Woods 2012 [31]**

| **Entry** | **Support for Judgement** | **Judgement** |
| --- | --- | --- |
| **Random Sequence Generation** | **Quote:** “Randomisation was completed using a dynamic allocation method”  **Comment:** Unclear how this was done. | **Unclear risk.** |
| **Allocation Concealment** | **Quote:** “By undertaking a complete list randomisation for each wave at each centre, allocation knowledge of the next assignment would be irrelevant as all participants for a centre would be randomized together.”  **Comment:** Probably true. | **Low risk.** |
| **Blinding of Participants and Personnel** | **Quote:** “Though participants could not be  blinded to their allocated treatment,”  **Comment:** Probably true. | **High risk.** |
| **Blinding of outcome assessment** (Hospitalisation) | **Quote:** “Though participants could not be  blinded to their allocated treatment, all follow-up data were gathered by blinded interviewers. In order to reduce the risk of participants occasionally and inadvertently informing researchers of the treatment they were receiving, explicit reminders were given to participants before assessment visits, and self-report measures were used wherever feasible. Assessors were also asked to record their impression of the arm to which each participant belonged, and their confidence in that prediction, so that any bias could be detected.”  **Comment:** Probably true. | **Low risk.** |
| **Incomplete outcome data addressed** (Hospitalisation) | **Quote:** Presented data is only in terms of rate, not absolute percentage. Only 69% of randomised participants had hospitalisation data.  **Comment:** Limited data. | **High risk.** |
| **Selective reporting** | **Quote:** The paper reports all outcomes highlighted in the trial registration [32].  **Comment:** Probably true. | **Low risk.** |

# **Appendix C: Reasons for exclusion**

No data for hospitalisation [33-70]

Ongoing Studies [71-87]

Not randomised control trials [88-91]

Subjects already hospitalised [92-94]

No dementia diagnosis [95]

Unable to analyse data by dementia status [96-98]

Study did not take place [99]

Intervention not aimed at dementia patients [100]

# **Appendix D Supplementary References**

1. Bass DMJ, K. S. Maslow, K. Wilson, N. L. Morgan, R. O. McCarthy, C. A. Looman, W. J. Snow, A. L. Kunik, M. E. Impact of the care coordination program "partners in Dementia Care" on veterans' hospital admissions and emergency department visits. Alzheimer's and Dementia: Translational Research and Clinical Interventions. 2015;1(1):13-22. doi: 10.1016/j.trci.2015.03.003.

2. Clinicaltrials.gov [Internet]. Bethesda (MD): National Library of Medicine (US). 2000 Feb 29. NCT00291161. Partners in Dementia Care: A Telephone Care Consultation Intervention Provided to Veterans in Partnership With Local Alzheimer's Association Chapters (PDC). 2006. Available from: <https://clinicaltrials.gov/ct2/show/NCT00291161>

3. Bass DMJ, K. S. Snow, A. L. Wilson, N. L. Morgan, R. Looman, W. J. McCarthy, C. A. Maslow, K. Moye, J. A. Randazzo, R. Garcia-Maldonado, M. Elbein, R. Odenheimer, G. Kunik, M. E. Caregiver outcomes of partners in dementia care: effect of a care coordination program for veterans with dementia and their family members and friends. Journal of the American Geriatrics Society. 2013;61(8):1377-86. doi: 10.1111/jgs.12362.

4. Morgan RO, Bass DM, Judge KS, Liu CF, Wilson N, Snow AL, et al. A break-even analysis for dementia care collaboration: Partners in Dementia Care. Journal of general internal medicine. 2015;30:804-9. doi: 10.1007/s11606-015-3205-x.

5. Bass DMJ, K. S. Snow, A. L. Wilson, N. L. Morgan, R. O. Maslow, K. Randazzo, R. Moye, J. A. Odenheimer, G. L. Archambault, E. Elbein, R. Pirraglia, P. Teasdale, T. A. McCarthy, C. A. Looman, W. J. Kunik, M. E. A controlled trial of Partners in Dementia Care: Veteran outcomes after six and twelve months. Alzheimer's Research and Therapy. 2014;1 6:9. doi: 10.1186/alzrt242.

6. Bellantonio SK, A. M. Fortinsky, R. H. Kleppinger, A. Robison, J. Gruman, C. Kulldorff, M. Trella, P. M. Efficacy of a geriatrics team intervention for residents in dementia-specific assisted living facilities: effect on unanticipated transitions. Journal of the American Geriatrics Society. 2008;56(3):523-8. doi: 10.1111/j.1532-5415.2007.01591.x.

7. Callahan CMB, M. A. Unverzagt, F. W. Austrom, M. G. Damush, T. M. Perkins, A. J. Fultz, B. A. Hui, S. L. Counsell, S. R. Hendrie, H. C. Effectiveness of collaborative care for older adults with Alzheimer disease in primary care: a randomized controlled trial. Jama. 2006;295(18):2148-57. doi: 10.1001/jama.295.18.2148.

8. Clinicaltrials.gov [Internet]. Bethesda (MD): National Library of Medicine (US). 2000 Feb 29. NCT00246896. Care Management for Patients With Alzheimer Disease and Their Family Caregivers. 2005. Available from: <https://clinicaltrials.gov/ct2/show/NCT00246896>

9. Duru OKE, S. L. Vassar, S. D. Chodosh, J. Vickrey, B. G. Cost evaluation of a coordinated care management intervention for dementia. American Journal of Managed Care. 2009;15(8):521-8.

10. Vickrey BGM, B. S. Connor, K. I. Pearson, M. L. Della Penna, R. D. Ganiats, T. G. Demonte, R. W., Jr. Chodosh, J. Cui, X. Vassar, S. Duan, N. Lee, M. The effect of a disease management intervention on quality and outcomes of dementia care: a randomized, controlled trial. Annals of Internal Medicine. 2006;145(10):713-26.

11. Eloniemi-Sulkava US, M. Laakkonen, M. Pietilä, M. Savikko, N. Kautiainen, H. Tilvis, R. S. Pitkälä, K. H. Family care as collaboration: effectiveness of a multicomponent support program for elderly couples with dementia. Randomized controlled intervention study. Journal of the American Geriatrics Society. 2009;57(12):2200-8. doi: 10.1111/j.1532-5415.2009.02564.x.

12. Engedal K. Day care for demented patients in general nursing homes. Effects on admissions to institutions and mental capacity. Scand J Prim Health Care. 1989;7(3):161-6.

13. Graff MJA, E. M. Vernooij-Dassen, M. J. Dekker, J. Jonsson, L. Thijssen, M. Hoefnagels, W. H. Rikkert, M. G. Community occupational therapy for older patients with dementia and their care givers: cost effectiveness study. Bmj. 2008;336(7636):134-8. doi: 10.1136/bmj.39408.481898.BE.

14. Graff MJV-D, M. J. Thijssen, M. Dekker, J. Hoefnagels, W. H. Rikkert, M. G. Community based occupational therapy for patients with dementia and their care givers: randomised controlled trial. Br Med J. 2006;333(7580):1196.

15. Kohler LM-F, C. Hein, J. Fendrich, K. Heymann, R. Thyrian, J. R. Hoffmann, W. Does an interdisciplinary network improve dementia care? Results from the IDemUck-study. Current Alzheimer Research. 2014;11(6):538-48.

16. Laakkonen MLK, H. Holtta, E. Savikko, N. Tilvis, R. S. Strandberg, T. E. Pitkala, K. H. Effects of Self-Management Groups for People with Dementia and Their Spouses - Randomized Controlled Trial. Journal of the American Geriatrics Society. 2016;64(4):752-60. doi: 10.1111/jgs.14055.

17. Laakkonen M-LH, Eeva H. Savikko, Niina Strandberg, Timo E. Suominen, Merja Pitkälä, Kaisu H. Psychosocial group intervention to enhance self-management skills of people with dementia and their caregivers: study protocol for a randomized controlled trial. Trials. 2012;13(1):133-. doi: 10.1186/1745-6215-13-133.

18. Meeuwsen EM, R. van der Aa, G. Goluke-Willemse, G. de Leest, B. van Raak, F. Scholzel-Dorenbos, C. Verheijen, D. Verhey, F. Visser, M. Wolfs, C. Adang, E. Rikkert, M. O. Cost-Effectiveness of One Year Dementia Follow-Up Care by Memory Clinics or General Practitioners: Economic Evaluation of a Randomised Controlled Trial. Plos One. 2013;8(11). doi: 10.1371/journal.pone.0079797.

19. Meeuwsen EJG, P. Melis, R. J. Adang, E. M. Goluke Willemse, G. A. Krabbe, P. F. De Leest, B. J. Van Raak, F. H. Scholzel Dorenbos, C. J. Visser, M. C. Wolfs, C. A. Vliek, S. Olde Rikkert, M. G. Cost-effectiveness of post-diagnosis treatment in dementia coordinated by multidisciplinary memory clinics in comparison to treatment coordinated by general practitioners: an example of a pragmatic trial. Journal of Nutrition, Health & Aging. 2009;13(3):242-8.

20. Clinicaltrials.gov [Internet]. Bethesda (MD): National Library of Medicine (US). 2000 Feb 29. NCT00554047. Comparison of Effectiveness and Costs of Post-diagnosis Treatment in Dementia (AD-Euro). 2007. Available from: <https://clinicaltrials.gov/ct2/show/NCT00554047>

21. Pitkala KHP, M. M. Laakkonen, M. L. Tilvis, R. S. Savikko, N. Kautiainen, H. Strandberg, T. E. Effects of the Finnish Alzheimer disease exercise trial (FINALEX): a randomized controlled trial. JAMA Internal Medicine. 2013;173(10):894-901. doi: 10.1001/jamainternmed.2013.359.

22. Pitkala KHR, M. M. Laakkonen, M. L. Tilvis, R. S. Kautiainen, H. Strandberg, T. E. Exercise rehabilitation on home-dwelling patients with Alzheimer's disease - a randomized, controlled trial. Study protocol. Trials. 2010;11:92. doi: 10.1186/1745-6215-11-92.

23. Sogaard RS, J. Waldorff, F. B. Eckermann, A. Buss, D. V. Phung, K. T. Waldemar, G. Early psychosocial intervention in Alzheimer's disease: cost utility evaluation alongside the Danish Alzheimer's Intervention Study (DAISY). BMJ open. 2014;4(1):e004105. doi: 10.1136/bmjopen-2013-004105.

24. Waldorff FBB, D. V. Eckermann, A. Rasmussen, M. L. Keiding, N. Rishoj, S. Siersma, V. Sorensen, J. Sorensen, L. V. Vogel, A. Waldemar, G. Efficacy of psychosocial intervention in patients with mild Alzheimer's disease: the multicentre, rater blinded, randomised Danish Alzheimer Intervention Study (DAISY). Bmj. 2012;345:e4693. doi: 10.1136/bmj.e4693.

25. Waldemar GW, F. B. Buss, D. V. Eckermann, A. Keiding, N. Rishoj, S. Siersma, V. Sorensen, J. Sorensen, L. V. Vogel, A. The Danish Alzheimer Intervention Study: Rationale, Study Design and Baseline Characteristics of the Cohort. Neuroepidemiology. 2011;36(1):52-61. doi: 10.1159/000322942.

26. Schwarzkopf LM, P. Kunz, S. Holle, R. Lauterberg, J. Marx, P. Mehlig, H. Wunder, S. Leidl, R. Donath, C. Graessel, E. Costs of care for dementia patients in community setting: an analysis for mild and moderate disease stage. Value in Health. 2011;14(6):827-35. doi: 10.1016/j.jval.2011.04.005.

27. Holle RG, E. Ruckdaschel, S. Wunder, S. Mehlig, H. Marx, P. Pirk, O. Butzlaff, M. Kunz, S. Lauterberg, J. Dementia care initiative in primary practice: study protocol of a cluster randomized trial on dementia management in a general practice setting. BMC Health Services Research. 2009;9:91. doi: 10.1186/1472-6963-9-91.

28. Donath CG, E. Grossfeld-Schmitz, M. Menn, P. Lauterberg, J. Wunder, S. Marx, P. Ruckdaschel, S. Mehlig, H. Holle, R. Effects of general practitioner training and family support services on the care of home-dwelling dementia patients--results of a controlled cluster-randomized study. BMC health services research. 2010;10:314.

29. Voigt-Radloff SG, M. Leonhart, R. Schornstein, K. Jessen, F. Bohlken, J. Metz, B. Fellgiebel, A. Dodel, R. Eschweiler, G. Vernooij-Dassen, M. Rikkert, M. O. Hull, M. A multicentre RCT on community occupational therapy in Alzheimer's disease: 10 sessions are not better than one consultation. Bmj Open. 2011;1(1). doi: 10.1136/bmjopen-2011-000096.

30. Voigt-Radloff S, Graff M, Leonhart R, Schornstein K, Vernooij-Dassen M, Olde-Rikkert M, et al. WHEDA study: Effectiveness of occupational therapy at home for older people with dementia and their caregivers - the design of a pragmatic randomised controlled trial evaluating a Dutch programme in seven German centres. BMC Geriatrics. 2009;9:44. doi: 10.1186/1471-2318-9-44.

31. Woods RB, E. Edwards, R. Elvish, R. Hoare, Z. Hounsome, B. Keady, J. Moniz-Cook, E. Orgeta, V. Orrell, M. Rees, J. Russell, I. REMCARE: reminiscence groups for people with dementia and their family caregivers - effectiveness and cost-effectiveness pragmatic multicentre randomised trial. Health Technology Assessment. 2012;16(50):1-121. doi: 10.3310/hta16480.

32. Woods RTB, E. Edwards, R. T. Hounsome, B. Keady, J. Moniz-Cook, E. D. Orrell, M. Russell, I. T. Reminiscence groups for people with dementia and their family carers: pragmatic eight-centre randomised trial of joint reminiscence and maintenance versus usual treatment: a protocol. Trials. 2009;10:64. doi: 10.1186/1745-6215-10-64.

33. Amieva HD, J. F. ETNA3, a clinical randomized study assessing three cognitive-oriented therapies in dementia: Rationale and general design. Revue Neurologique. 2013;169(10):752-6. doi: 10.1016/j.neurol.2013.07.011.

34. Amieva HD, J. F. Etna3, a clinical randomized study assessing 3 cognitive-oriented therapies in alzheimer's disease. Journal of Nutrition, Health and Aging. 2013;17 (9):802-3.

35. Amieva HR, P. H. Grandoulier, A. S. Meillon, C. De Rotrou, J. Andrieu, S. Berr, C. Desgranges, B. Dubois, B. Girtanner, C. Joel, M. E. Lavallart, B. Nourhashemi, F. Pasquier, F. Rainfray, M. Touchon, J. Chene, G. Dartigues, J. F. Group and individual cognitive therapies in Alzheimer's disease: The ETNA3 randomized trial. International Psychogeriatrics. 2016;28(5):707-17. doi: 10.1017/S1041610215001830.

36. Current Controlled Trials [Internet]. London: BioMed Central. [date unknown]. ISRCTN15609358. Evaluation of emotion-oriented care versus usual care for elderly persons with dementia in the nursing home. 2009. Available from: <http://isrctn.com/ISRCTN15609358>

37. Serrani Azcurra DJL. A reminiscence program intervention to improve the quality of life of long-term care residents with Alzheimer's disease: a randomized controlled trial. Revista brasileira de psiquiatria (Sao Paulo, Brazil : 1999). 2012;34:422-33.

38. Beer CD, Horner B, Almeida OP, Scherer S, Lautenschlager NT, Bretland N, et al. Dementia in residential care: education intervention trial (DIRECT); protocol for a randomised controlled trial. Trials. 2010;11:63. doi: 10.1186/1745-6215-11-63.

39. Chenoweth L. The percen study: Supporting client and care outcomes in the residential dementia care setting. Alzheimer's and Dementia. 2011;1):S292. doi: 10.1016/j.jalz.2011.05.847.

40. Chenoweth L, King MT, Jeon Y-H, Brodaty H, Stein-Parbury J, Norman R, et al. Caring for Aged Dementia Care Resident Study (CADRES) of person-centred care, dementia-care mapping, and usual care in dementia: a cluster-randomised trial. The Lancet Neurology. 2009;8:317-25. doi: 10.1016/S1474-4422(09)70045-6.

41. Eloniemi-Sulkava UN, I. Hentinen, M. Kivelä, S. Sivenius, J. Sulkava, R. Effects of supporting community-living demented patients and their caregivers: a randomized trial. Journal of the American Geriatrics Society. 2001;49(10):1282-7. doi: 10.1046/j.1532-5415.2001.49255.x.

42. Eloniemi-Sulkava UN, Irma-Leena Hentinen, Maria Kivela, Sirkka-Liisa Sivenius, Juhani Sulkava, Raimo. Supportive community living in demented patients and their caregivers: A randomized trial. Zeitschrift fur Gerontopsychologie und psychiatrie. 2004;17(1):31-40. doi: 10.1024/1011-6877.17.1.31.

43. Eloniemi-Sulkava UP, K. Saarenheimo, M. Pietila, M. Virtanen, L. Laakkonen, M. L. Huusko, T. Tilvis, R. Supporting demented patients and their spouse caregivers in community care randomized, controlled intervention study in 2004-2006. International Psychogeriatrics. 2005;17:338-.

44. Eloniemi-Sulkava UR, T. A dementia co-ordinator supports home care of dementia patients. Sairaanhoitaja. 2004;77(4):17-9.

45. Holle DR, Martina Buscher, Ines Reuther, Sven Müller, René Halek, Margareta. Process evaluation of the implementation of dementia-specific case conferences in nursing homes (FallDem): study protocol for a randomized controlled trial. Trials. 2014;15(1):485-. doi: 10.1186/1745-6215-15-485.

46. Jansen APDvH, Hein P. J. Nijpels, Giel Rijmen, Frank Dröes, Rose-Marie Pot, Anne-Margriet Schellevis, François G. Stalman, Wim A. B. van Marwijk, Harm W. J. Effectiveness of case management among older adults with early symptoms of dementia and their primary informal caregivers: A randomized clinical trial. International Journal of Nursing Studies. 2011;48(8):933-43. doi: 10.1016/j.ijnurstu.2011.02.004.

47. Koivisto AMH, I. Valimaki, T. H. Hongisto, H. K. Hiltunen, A. Karppi, P. Sivenius, J. Soininen, H. Martikainen, J. A. Efficacy of early psychosocial intervention for persons with Alzheimer's disease and caregivers: The Kuopio ALSOVA study results, 3-year follow-up. European Geriatric Medicine. 2014;5:S66.

48. Koivisto AV, T. Martikainen, J. Early psychosocial intervention did not delay institutionalization in patients with Alzheimer's disease: Alsova study reports. Alzheimer's and Dementia. 2013;1):P527. doi: 10.1016/j.jalz.2013.04.262.

49. MacNeil Vroomen JB, J. E. van de Ven, P. M. Joling, K. J. van Mierlo, L. D. Meiland, F. J. Moll van Charante, E. P. van Hout, H. P. de Rooij, S. E. Community-dwelling patients with dementia and their informal caregivers with and without case management: 2-year outcomes of a pragmatic trial. Journal of the American Medical Directors Association. 2015;16(9):800.e1-8. doi: 10.1016/j.jamda.2015.06.011.

50. Martikainen JAK, H. Vaatainen, S. Valimaki, T. Hongisto, K. Hallikainen, I. Sivenius, J. Soininen, H. Koivisto, A. Effectiveness of the early psychosocial intervention on institutionalization of patients with mild Alzheimer's disease and caregivers' quality of life - An alsova study. Value in Health. 2013;16 (7):A618-A9. doi: 10.1016/j.jval.2013.08.1803.

51. Moyle W, Beattie E, Draper B, Shum D, Thalib L, Jones C, et al. Effect of an interactive therapeutic robotic animal on engagement, mood states, agitation and psychotropic drug use in people with dementia: a cluster-randomised controlled trial protocol: Table 1. BMJ Open. 2015;5:e009097. doi: 10.1136/bmjopen-2015-009097.

52. Clinicaltrials.gov [Internet]. Bethesda (MD): National Library of Medicine (US). 2000 Feb 29. NCT01796314. Impact of a Therapeutic Educational Programme for Alzheimer's Disease Patients and Their Caregiver in Community Dwelling, on the Ad Patient's Quality of Life. 2013. Available from: <https://clinicaltrials.gov/ct2/show/NCT01796314>

53. Clinicaltrials.gov [Internet]. Bethesda (MD): National Library of Medicine (US). 2000 Feb 29. NCT01280890. Person-centred care and dementia care mapping among nursing home patients - a 10 months randomised controlled intervention study. 2011. Available from: <Http://clinicaltrials.gov/show/NCT01280890>

54. Nourhashemi FA, S. Gillette-Guyonnet, S. Giraudeau, B. Cantet, C. Coley, N. Vellas, B. Plasa Grp. Effectiveness of a specific care plan in patients with Alzheimer's disease: cluster randomised trial (PLASA study). British Medical Journal. 2010;340. doi: 10.1136/bmj.c2466.

55. Nourhashemi F, Gillette-Guyonnet S, Andrieu S, Rolland Y, Ousset P-J, Vellas B, et al. A randomized trial of the impact of a specific care plan in 1120 Alzheimer's patients (PLASA Study) over a two-year period: design and baseline data. The journal of nutrition, health & aging. 2008;12:263-71.

56. Prick A-EdL, Jacomine Scherder, Erik Pot, Anne Margriet. Home-based exercise and support programme for people with dementia and their caregivers: study protocol of a randomised controlled trial. BMC Public Health. 2011;11(1):894-.

57. Saez IO, M. Ciudad, M. J. Fort, I. Llopart, J. R. Haritou, M. Alladin project: A technology platform for the assisted living of dementia elderly individuals and their carers. European Geriatric Medicine. 2011;2:S151-S2. doi: 10.1016/j.eurger.2011.06.002.

58. Wells YJ, A. F. Evaluation of a special nursing home unit for dementia sufferers: a randomised controlled comparison with community care. Australian & New Zealand Journal of Psychiatry. 1987;21(4):524-31.

59. Kallio E-L, Öhman H, Hietanen M, Soini H, Strandberg TE, Kautiainen H, et al. Effects of Cognitive Training on Cognition and Quality of Life of Older Persons with Dementia. Journal of the American Geriatrics Society. 2018;66(4):664-70. doi: 10.1111/jgs.15196.

60. Australian New Zealand Clinical Trials Registry [Internet]. Sydney (NSW): NHMRC Clinical Trials Centre, University of Sydney (Australia); 2005. ACTRN12614000976684. Cognitive training on people with dementia - a randomized controlled trial. 2014. Available from: <http://www.anzctr.org.au/ACTRN12614000976684.aspx>

61. Chodosh JC, K. Vassar, S. Pearson, M. Lee, M. Mittman, B. Ganz, D. Vickrey, B. Implementing dementia care management in a medicare managed care plan: A randomized controlled trial. Journal of the American Geriatrics Society. 2015;63:S1. doi: 10.1111/jgs.13439.

62. Chiatti CM, F. Rimland, J. M. Cherubini, A. Scarpino, O. Spazzafumo, L. Lattanzio, F. The UP-TECH project, an intervention to support caregivers of Alzheimer's disease patients in Italy: Study protocol for a randomized controlled trial. Trials. 2013;14 (1)(155). doi: 10.1186/1745-6215-14-155.

63. Chiatti CR, J. M. Bonfranceschi, F. Masera, F. Bustacchini, S. Cassetta, L. The UP-TECH project, an intervention to support caregivers of Alzheimer's disease patients in Italy: preliminary findings on recruitment and caregiving burden in the baseline population. Aging & mental health. 2015;19(6):517-25. doi: 10.1080/13607863.2014.954526.

64. Chien WTL, I. Y. Randomized controlled trial of a dementia care programme for families of home-resided older people with dementia. Journal of Advanced Nursing. 2011;67(4):774-87. doi: 10.1111/j.1365-2648.2010.05537.x.

65. Chodosh JC, B. A. Connor, K. I. Cope, D. W. Liu, H. S. Ganz, D. A. Richman, M. J. Cherry, D. L. Blank, J. M. Carbone, R. D. Wolf, S. M. Vickrey, B. G. Dementia Care Management in an Underserved Community: The Comparative Effectiveness of Two Different Approaches. Journal of Aging and Health. 2015;27(5):864-93. doi: 10.1177/0898264315569454.

66. Ven GD, I. Herpen, E. Koopmans, Rtcm Donders, R. Zuidema, S. U. Adang, E. M. M. Vernooij-Dassen, Mjfj. The economics of dementia-care mapping in nursing homes: A cluster-randomised controlled trial. PloS one [Internet]. 2014; 9(1). Available from: <http://onlinelibrary.wiley.com/o/cochrane/clcentral/articles/371/CN-01051371/frame.html>.

67. Villars HG, V. Perrin, A. Hein, C. Elmalem, S. De Peretti, E. Zueras, A. Vellas, B. Nourhashemi, F. Study protocol: Randomised controlled trial to evaluate the impact of an educational programme on Alzheimer's disease patients' quality of life. Alzheimer's Research and Therapy. 2014;6 (no pagination)(66). doi: 10.1186/s13195-014-0066-1.

68. Wolfs CAD, C. D. Severens, J. L. Kessels, A. Verkaaik, M. Verhey, F. R. [The costs and benefits of an integrated approach to dementia]. Tijdschr Psychiatr. 2011;53(9):657-65.

69. Zamrini EA, T. Foster, N. Proactive dementia care: First-year findings. Alzheimer's and Dementia. 2012;1):P599-P600. doi: 10.1016/j.jalz.2012.05.2085.

70. Lamb SE, Sheehan B, Atherton N, Nichols V, Collins H, Mistry D, et al. Dementia And Physical Activity (DAPA) trial of moderate to high intensity exercise training for people with dementia: randomised controlled trial. Bmj. 2018;361:k1675. doi: 10.1136/bmj.k1675.

71. Agar MB, E. Luckett, T. Phillips, J. Luscombe, G. Goodall, S. Mitchell, G. Pond, D. Davidson, P. M. Chenoweth, L. Pragmatic cluster randomised controlled trial of facilitated family case conferencing compared with usual care for improving end of life care and outcomes in nursing home residents with advanced dementia and their families: the IDEAL study protocol. BMC Palliat Care. 2015;14:63. doi: 10.1186/s12904-015-0061-8.

72. Leroi I, Woolham J, Gathercole R, Howard R, Dunk B, Fox C, et al. Does telecare prolong community living in dementia? A study protocol for a pragmatic, randomised controlled trial. Trials. 2013;14:349. doi: 10.1186/1745-6215-14-349.

73. Wenborn JH, S. Moniz-Cook, E. Mountain, G. Poland, F. King, M. Omar, R. Morris, S. Vernooij-Dassen, M. Challis, D. Michie, S. Russell, I. Sackley, C. Graff, M. O'Keeffe, A. Crellin, N. Orrell, M. Community occupational therapy for people with dementia and family carers (COTiD-UK) versus treatment as usual (Valuing Active Life in Dementia [VALID] programme): Study protocol for a randomised controlled trial. Trials. 2016;17 (1) (no pagination)(65). doi: 10.1186/s13063-015-1150-y.

74. Malmgren Fänge A, Schmidt SM, Nilsson MH, Carlsson G, Liwander A, Dahlgren Bergström C, et al. The TECH@HOME study, a technological intervention to reduce caregiver burden for informal caregivers of people with dementia: study protocol for a randomized controlled trial. Trials. 2017;18(1):63. doi: 10.1186/s13063-017-1796-8.

75. Fortinsky RH, Gitlin LN, Pizzi LT, Piersol CV, Grady J, Robison JT, et al. Translation of the Care of Persons with Dementia in their Environments (COPE) intervention in a publicly-funded home care context: rationale and research design. Contemporary clinical trials. 2016;49:155‐65. doi: 10.1016/j.cct.2016.07.006.

76. Australian New Zealand Clinical Trials Registry [Internet]. Sydney (NSW): NHMRC Clinical Trials Centre, University of Sydney (Australia); 2005. ACTRN12616000927426p. Efficacy of the 'Support for Life' program on improving quality of life for people with dementia or cognitive decline and their families or carers. 2016. Available from: <http://www.anzctr.org.au/ACTRN12616000927426.aspx>

77. Surr CAW, Rebecca E. A. Lilley-Kelly, Amanda Cicero, Robert Meads, David Ballard, Clive Burton, Kayleigh Chenoweth, Lynn Corbett, Anne Creese, Byron Downs, Murna Farrin, Amanda J. Fossey, Jane Garrod, Lucy Graham, Elizabeth H. Griffiths, Alys Holloway, Ivana Jones, Sharon Malik, Baber Siddiqi, Najma. Evaluating the effectiveness and cost-effectiveness of Dementia Care Mapping™ to enable person-centred care for people with dementia and their carers (DCM-EPIC) in care homes: study protocol for a randomised controlled trial. Trials. 2016;17:1-17. doi: 10.1186/s13063-016-1416-z.

78. Current Controlled Trials [Internet]. London: BioMed Central. [date unknown]. ISRCTN82288852. Evaluating the effectiveness and cost effectiveness of Dementia Care Mapping (DCM) to enable person-centred care for people with dementia and their carers. 2014. Available from: <http://www.isrctn.com/ISRCTN82288852>

79. Knapp MT, L. Patel, A. Spector, A. Hallam, A. Woods, B. Orrell, M. Cognitive stimulation therapy for people with dementia: cost-effectiveness analysis. British Journal of Psychiatry. 2006;188:574-80.

80. Tuijt R, Livingston G, Gould RL, Jones R, Verdaguer ES, Orgeta V. IDEA intervention to prevent depressive symptoms and promote well-being in early-stage dementia: Protocol for a randomised controlled feasibility study. BMJ Open. 2018;8 (2) (no pagination)(e021074). doi: 10.1136/bmjopen-2017-021074.

81. Rhee J, Meller A, Krysinska K, Gonski P, Naganathan V, Zwar N, et al. Advance care planning for patients with advanced illnesses attending hospital outpatient clinics study: a study protocol for a randomised controlled trial. BMJ Open. 2019;9(1). doi: 10.1136/bmjopen-2018-023107.

82. Australian New Zealand Clinical Trials Registry [Internet]. Sydney (NSW): NHMRC Clinical Trials Centre, University of Sydney (Australia); 2005. ACTRN12617000280303. Effect of Advance Care Planning (ACP) on health resources utilisation and quality of care for patients with advanced illnesses attending hospital outpatient clinics. 2017. Available from: <https://www.anzctr.org.au/Trial/Registration/TrialReview.aspx?id=372002>

83. Mor V, Volandes AE, Gutman R, Gatsonis C, Mitchell SL. PRagmatic trial Of Video Education in Nursing homes: The design and rationale for a pragmatic cluster randomized trial in the nursing home setting. Clinical Trials. 2017;14(2):140-51. doi: 10.1177/1740774516685298.

84. Goeman D, Comans T, Enticott JC, Renehan E, Beattie E, Kurrle S, et al. Evaluating the Efficacy of the “Support for Life” Program for People with Dementia and Their Families and Carers’ to Enable Them to Live Well: A Protocol for a Cluster Stepped Wedge Randomized Controlled Trial. Frontiers in Public Health. 2016;4. doi: 10.3389/fpubh.2016.00245.

85. Netherlands Trial Register [Internet]. Amsterdam: Academic Medical Center (The Netherlands). 2004 Oct 26. NTR5936. Adaptive Implementation and Validation of the positively evaluated Meeting Centers Support Programme for people with dementia and their carers in Europe. 2016. Available from: <https://www.trialregister.nl/trial/5580>

86. Netherlands Trial Register [Internet]. Amsterdam: Academic Medical Center (The Netherlands). 2004 Oct 26. NTR6581. Hospital at Home care for older patients with cognitive impairment: a protocol for a randomized controlled feasibility trial. 2017. Available from: <https://www.trialregister.nl/trial/6406>

87. Australian New Zealand Clinical Trials Registry [Internet]. Sydney (NSW): NHMRC Clinical Trials Centre, University of Sydney (Australia); 2005. ACTRN12618000600246. Optimising functional independence of older persons with dementia: Evaluation of the Interdisciplinary Home-bAsed Reablement Program (I-HARP). 2018. Available from: <http://www.anzctr.org.au/ACTRN12618000600246.aspx>

88. ClinicalTrials.gov [Internet]. Bethesda (MD): National Library of Medicine (US). 2000 Feb 29. NCT01943071. Effects and Costs of a Day Care Centre Program Designed for People With Dementia. 2013. Available from: <https://ClinicalTrials.gov/show/NCT01943071>

89. Phelan EAD, K. J. Anderson, L. A. Owens, S. B. A systematic review of intervention studies to prevent hospitalizations of community-dwelling older adults with dementia. Med Care. 2015;53(2):207-13. doi: 10.1097/MLR.0000000000000294.

90. Health Technology Assessment Database [Internet]. A pragmatic randomised controlled trial to evaluate the effectiveness and cost effectiveness of Collaborative cARE for people with DEMentia in primary care (CARE-DEM trial) (Project record). 2011. Available from: <http://www.crd.york.ac.uk/crdweb/ShowRecord.asp?LinkFrom=OAI&ID=32011000675>

91. Morrin H, Fang T, Servant D, Aarsland D, Rajkumar AP. Systematic review of the efficacy of non-pharmacological interventions in people with Lewy body dementia. International Psychogeriatrics. 2018;30(3):395-407. doi: 10.1017/S1041610217002010.

92. Sampson ELJ, L. Thune-Boyle, I. C. Kukkastenvehmas, R. King, M. Leurent, B. Tookman, A. Blanchard, M. R. Palliative assessment and advance care planning in severe dementia: an exploratory randomized controlled trial of a complex intervention. Palliative Medicine. 2011;25(3):197-209. doi: 10.1177/0269216310391691.

93. Tibaldi VA, N. Ponzetto, M. Stasi, M. F. Amati, D. Raspo, S. Roglia, D. Molaschi, M. Fabris, F. A randomized controlled trial of a home hospital intervention for frail elderly demented patients: behavioral disturbances and caregiver's stress. Arch Gerontol Geriatr Suppl. 2004;(9):431-6.

94. Clinicaltrials.gov [Internet]. Bethesda (MD): National Library of Medicine (US). 2000 Feb 29. NCT02388711. A Trial of the C-Trac Intervention for Dementia Patients. 2015. Available from: <https://clinicaltrials.gov/ct2/show/NCT02388711>

95. Netherlands Trial Register [Internet]. Amsterdam: Academic Medical Center (The Netherlands). 2004 Oct 26. NTR66. Effectiveness and cost-effectiveness of a care-programme by district nurses among elderly with dementia symptoms and their primary informal caregiver. 2002. Available from: <https://www.trialregister.nl/trial/39>

96. Bass DMC, P. A. Looman, W. J. McCarthy, C. A. Eckert, S. The Cleveland Alzheimer's managed care demonstration: outcomes after 12 months of implementation. Gerontologist. 2003;43(1):73-85.

97. Brooker DJA, E. Scally, A. J. Clancy, D. The enriched opportunities programme for people with dementia: a cluster-randomised controlled trial in 10 extra care housing schemes. Aging & Mental Health. 2011;15(8):1008-17. doi: 10.1080/13607863.2011.583628.

98. Kiosses DN, Ravdin LD, Gross JJ, Raue P, Kotbi N, Alexopoulos GS. Problem adaptation therapy for older adults with major depression and cognitive impairment: a randomized clinical trial. JAMA Psychiatry. 2015;72:22-30. doi: 10.1001/jamapsychiatry.2014.1305.

99. Current Controlled Trials [Internet]. London: BioMed Central. [date unknown]. ISRCTN33790007. The DAPE study: the impact of dementia advisors on the quality of life of dementia sufferers. 2009. Available from: <http://isrctn.com/ISRCTN33790007>

100. Hanson LC, Zimmerman S, Song M-K, Lin F-C, Rosemond C, Carey TS, et al. Effect of the Goals of Care Intervention for Advanced Dementia. JAMA Internal Medicine. 2017;177(1). doi: 10.1001/jamainternmed.2016.7031.
